# Nursing Students Learn Vaccination Using Kahoot! Gamification: An Intervention Study of Knowledge, Satisfaction, Interest, and Collaboration

**DOI:** 10.1155/nrp/3518943

**Published:** 2025-07-28

**Authors:** Aeen Mohammadi, Sanaz Aazami, Akbar Azizifar

**Affiliations:** ^1^Department of Medical Education, Smart University of Medical Sciences, Tehran, Iran; ^2^Department of Nursing, Faculty of Nursing and Midwifery, Ilam University of Medical Science, Ilam, Iran; ^3^Department of English Language, Faculty of Medicine, Ilam University of Medical Sciences, Ilam, Iran

**Keywords:** collaborative learning, formative feedback, gamification, nursing students, vaccination

## Abstract

**Introduction:** Nursing students often lack engagement and confidence in vaccination procedures, despite their critical public health importance. To address this gap, this study aimed to assess the effect of Kahoot!-based gamification on nursing students' knowledge, satisfaction, interest, and collaboration regarding vaccination.

**Methods:** The students were randomly divided into two groups: case (Kahoot! game) and control (teaching in the traditional way). Eight different games were created based on vaccination topics. During each session, the case group played one of the games developed by Kahoot!. Then, at the end of 8 sessions, the posttest was taken from both controls and cases.

**Results:** Findings from ANCOVA showed that the average knowledge after the internship in the group that used gamification with Kahoot! was significantly (*F* [1, 69] = 27.208, *p* ≤ 0.001) increased even after controlling for the effect of baseline knowledge. An independent *t*-test was performed to compare the average satisfaction of the internship between the two groups of control (mean = 7.5, SD = 1.34) and intervention (mean = 8.7, SD = 1.04), which showed to be significant (*t* (70) = 4.23, *p* ≤ 0.001). In addition, our results showed that the overall average interest in Kahoot!'s classes was 34.1 (SD = 2.03), ranging from 29 to 38. The average score for level of interaction at Kahoot!'s classes was 33.47 (SD = 2.36), ranging from 26 to 37.

**Conclusions:** In general, our findings showed that nursing students' knowledge and satisfaction significantly increased after the end of 8 weeks of Kahoot! gamification. Moreover, we found that the level of interest and collaboration among students after gamification with Kahoot! was significantly higher than the average score of 2.5.

## 1. Introduction

Nursing is a dynamic profession that requires the seamless integration of theoretical knowledge with practical skills to ensure high-quality patient care. However, studies consistently highlight gaps in the readiness and practical competencies of newly graduated nurses [1, 2]. This shortfall underscores the need for innovative educational strategies that not only convey knowledge but also foster active engagement, critical thinking, and long-term retention of learning.

In recent years, the rapid advancement of technology has introduced novel, interactive teaching methods into nursing education, including simulation, virtual reality, and gamification [3, 4]. These active learning approaches aim to create stimulating environments that make learning more appealing and effective, particularly for today's digitally native students [5].

Gamification, defined as the integration of game mechanics into nongame contexts to achieve educational objectives, has emerged as a promising strategy to enhance student motivation, engagement, and learning outcomes [6, 7]. Unlike traditional didactic methods, gamification fosters teamwork, provides immediate feedback, and introduces a sense of competition [8, 9]. Meta-analyses have shown that gamification improves both cognitive and behavioral engagement across educational fields [10, 11].

Vaccination is one of the most critical components of public health, playing a key role in preventing infectious diseases and controlling outbreaks. Community health nursing programs worldwide place significant emphasis on vaccination as part of their curricula, given that nurses are at the frontline of immunization efforts [12]. Teaching vaccination involves not only theoretical instruction, covering topics such as immunization schedules, contraindications, and cold chain management, but also practical skill-building, including safe injection practices and patient counseling. However, studies indicate that many nursing students feel underprepared to administer vaccinations confidently and safely, underscoring the need for enhanced educational strategies [13]. Traditional methods, such as lectures and demonstrations, while widely used, may fall short in engaging students [14]. Therefore, the use of innovative methods such as gamification could address these gaps by increasing motivation, facilitating active learning and strengthening both knowledge and skills.

While gamification is increasingly used in medical and health sciences education, its application in nursing remains underexplored, particularly in relation to essential community health topics such as vaccination [15, 16]. Vaccination education is critical for nursing students, as they are key players in immunization programs that safeguard community health. Traditional teaching methods may fail to fully engage students or convey the complexity and importance of immunization practices [17].

Kahoot! is a widely used gamification platform that allows instructors to create interactive quizzes and games, promoting competition and collaboration among students [18]. Compared to other tools such as Quizlet or Socrative, Kahoot! has the added advantage of combining audiovisual stimulation with real-time feedback and leaderboard features, making it especially effective in capturing students' attention and encouraging participation [19, 20]. Despite its popularity, the use of Kahoot! specifically for teaching vaccination content to nursing students has not been rigorously studied. Understanding whether gamification can enhance knowledge, satisfaction, and collaboration in this context is vital for informing future curricular innovations. Therefore, the present study aims to assess the impact of gamification using the Kahoot! platform on nursing students' knowledge, satisfaction, interest, and collaboration regarding vaccination, addressing a significant gap in the literature and contributing to the development of engaging, effective nursing education practices.

## 2. Materials and Methods

This quasiexperimental study was conducted between September and December 2023 among undergraduate nursing students at Ilam University of Medical Sciences, Iran. The study aimed to evaluate the effect of gamification using Kahoot! on students' knowledge, satisfaction, interest, and collaboration during their community health internship.

### 2.1. Participants and Research Population

The study population included all third-semester undergraduate nursing students enrolled in the 2023 academic year who were undertaking their community health internship.

#### 2.1.1. Inclusion Criteria

✓ Third-semester undergraduate nursing students enrolled in the community health internship course during the Fall 2023 semester.
✓ Successfully completed the theoretical course in community health nursing (as verified by academic records).
✓ Provided informed oral consent to participate in the study.

#### 2.1.2. Exclusion Criteria

✓ Students with prior participation in formal gamified educational interventions related to vaccination.
✓ Students who were absent for more than 1 of the 8 internship sessions (to ensure exposure to the full intervention).

All invited students consented to participate, and there was no attrition during the study.

### 2.2. Sampling and Randomization

This study employed a cluster randomization method to minimize contamination between groups. The full cohort of 70 eligible third-semester undergraduate nursing students at Ilam University of Medical Sciences was included, and these students were already organized by the university into 10 internship groups, each consisting of 7 students, as part of the regular community health rotation schedule.

To allocate groups into control and intervention conditions, we used a computer-generated randomization sequence. Five groups (totaling 35 students) were assigned to the intervention group (Kahoot! gamification), and five groups (35 students) to the control group (routine instruction). Randomization was performed at the cluster (group) level rather than the individual level to avoid cross-group exposure and ensure practical feasibility within the clinical education setting. To prevent any potential data contamination between groups, the control group completed their full internship program prior to the start of the intervention group's Kahoot! sessions. Since student groups were scheduled in chronological order, there was no overlap in timing, ensuring that participants in the control group had no exposure to the Kahoot! content or discussion during the study period.

### 2.3. Intervention Details

According to the nursing curriculum in this respective university, students had to complete 12 practical sessions in health centers, which were divided into 10 groups of equal size. These groups were assigned to five health centers for practical training. Each health center hosted one group at a time in a sequential, nonoverlapping schedule. Using randomly generated numbers, five health centers were assigned to the intervention group (teaching using Kahoot! Gamification), and the other five were assigned as controls (routine teaching with no additional strategy). Therefore, 35 students from five different health centers were offered the gamification using Kahoot! platform, and 35 students take their routine internship program as usual.

Students in the control group participated in standard community health internship activities as outlined in the national nursing curriculum. These activities included direct observation and occasional supervised assistance in routine vaccination procedures conducted at the health centers. Students observed cold chain management practices such as proper vaccine storage, temperature monitoring of refrigerators, and preparation of vaccines before administration.

They also followed clinical staff during vaccination sessions for infants and children, learning about vaccine schedules, contraindications, informed consent procedures, and documentation in immunization records. Where permitted, students participated in patient counseling, including educating caregivers on postvaccination care and managing mild side effects such as fever or swelling. All learning occurred under the supervision of a community health nurse, with no use of digital or gamified tools. Instruction was delivered through in-person mentoring, observation, and question-and-answer exchanges in the clinical environment.

Twelve gamified quizzes were initially developed on various vaccination-related topics. These included the following: (1) Introducing the types of vaccines, how to create immunity in each type of vaccine, the cold storage chain, and the place of storage in the refrigerator; (2) vaccines' side effects, complications, and its management, essential care, and dosage of acetaminophen after vaccination; (3) method of injection, place of injection, and related points; (4) national vaccination program for children; (5) national vaccination program for children aged 1–6 years but having a time delay in receiving vaccines; (6) national vaccination program for children older than 7 years old with a time delay in receiving vaccines; (7) vaccination in special cases such as pregnant mothers, children with a history of receiving blood products, preterm children, and children with special nutritional needs; (8) allergy to DPT vaccine, diagnosis, and indications; (9) vaccination of children with symptoms of cold, fever, diarrhea, or other physical diseases: do and do not; (10) vaccines at birth: with an emphasis on the HBV vaccine and its do and do not; (11) vaccination of infants born from mothers with positive HIV; and (12) other vaccines, including yellow fever, meningitis, and influenza.

Due to adjustments in internship scheduling during the COVID-19 pandemic, the number of sessions was reduced from 12 to 8 in order to minimize student exposure to the virus. This policy was taken from the faculty to reduce the amount of students' exposure to the virus. Therefore, the preidentified games were merged together forming the final 8 sessions. The merged sessions are described as follows:

Sessions 3 and 4, Sessions 5 and 6, Sessions 8 and 9, and Sessions 11 and 12 were merged, and two separate games were played in one session.

No additional strategy other than routine approaches was offered to students of the control group during their internship.

Although hands-on practical skills were not directly evaluated in a clinical setting, the Kahoot! games were designed to assess applied knowledge relevant to vaccination practices. For example, one of the games asked students to identify the correct injection site, the appropriate needle angle, and whether a vaccine should be administered intramuscularly or subcutaneously.

### 2.4. Timeframe

The study spanned 12 weeks in total: 2 weeks for preparation and pretesting, 8 weeks of intervention delivery (September–November 2023), and 2 weeks for posttesting and final data collection in December 2023.

#### 2.4.1. Games

Based on the course content, a series of quizzes was developed using the Kahoot! platform, where students participated as players in interactive “games.” This format enabled the integration of core gamification elements, such as audio, video, point scoring, and immediate feedback, into an informal yet engaging assessment strategy. The quizzes were designed using various formats supported by Kahoot!, including multiple-choice (with four options), true/false, short-answer, slider scales, puzzles, and pin placement questions.

**Figure 1:**
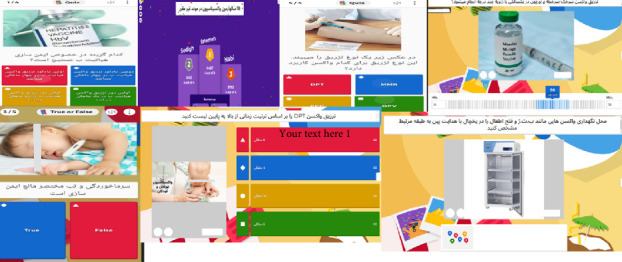
Screenshot of a Kahoot! game interface used during the intervention sessions.

Once instructors generated a session-specific PIN code, students accessed the games via their smartphones or web browsers. Multimedia features such as images and videos were embedded to enhance engagement, and instructors retained control over game speed and pacing (Figure 1). Points were awarded for each correct answer, with additional points granted for faster response times. To emphasize key content, selected questions were assigned double points. A live scoreboard was displayed after each item, fostering a competitive yet enjoyable atmosphere and motivating students to improve their ranking.

The decision to pause the game for discussion and feedback was at the discretion of the instructor. In practice, instructors facilitated short debriefs after nearly every question to provide immediate clarification and reinforce learning points. Upon completion of each session, the instructor provided a brief content review and summary to consolidate students' understanding and ensure effective knowledge transfer.

At the start of the first session, a pretest was administered to both the control and intervention groups. Throughout the intervention period, students in the experimental group engaged with one or two Kahoot! games per session. At the conclusion of the eighth session, a posttest was conducted for both groups. In addition, students in the intervention group completed standardized measures evaluating their levels of satisfaction, collaboration, and interest related to the gamified learning experience.

#### 2.4.2. Measurement

The studied outcomes were assessed using the following instruments: (1) a checklist made by the researcher to measure the level of knowledge regarding vaccination, (2) a standard questionnaire to measure collaboration and interest, and (3) a visual tool to measure satisfaction.

##### 2.4.2.1. Knowledge

A questionnaire was developed to assess students' knowledge of vaccination, based on the immunization guide approved by the National Immunization Committee [21]. The instrument contained 15 items: two were true/false questions assessing factual understanding, while the remaining 13 were multiple-choice questions with a single correct answer. Each correct answer was awarded one point, resulting in a total possible score ranging from 0 to 15. Higher scores indicated a greater level of knowledge regarding vaccination. To ensure the content validity of the developed questionnaire, five faculty members were invited to review and evaluate the items. The content validity index (CVI), as described by Waltz et al. [22], was used to assess the relevance, clarity, and simplicity of each item. Experts' feedback was incorporated into the final version of the questionnaire. The average CVI across all items was 0.89, indicating excellent content validity. To assess reliability, the questionnaire was administered to a group of 10 nursing students who were not part of the main study sample. The Cronbach's alpha coefficient was calculated as 0.84, which is considered acceptable for confirming the reliability of the instrument.

##### 2.4.2.2. Collaboration and Interest

To measure collaboration and interest, a standardized questionnaire designed to assess group dynamics in virtual medical education settings was used. McCoy [23] has used Cronbach's alpha coefficient to measure the reliability of this questionnaire, which showed 0.86 for collaboration and 0.88 for interaction. In addition, content validity was confirmed through expert evaluation [23].

The collaboration subscale includes eight items rated on a five-point Likert scale, ranging from 1 (*strongly disagree*) to 5 (*strongly agree*), resulting in a total score range from 8 to 40. Higher scores indicate greater levels of collaboration. Similarly, interest is assessed through eight items on the same Likert scale, with a total score also ranging from 8 to 40. Higher scores reflect higher levels of interest.

##### 2.4.2.3. Satisfaction

Students' satisfaction was measured using a Visual Analog Scale (VAS). This tool consists of a vertical line with two facial expressions at its lower and upper ends, representing complete dissatisfaction and complete satisfaction, respectively. Students marked a point along the line corresponding to their level of satisfaction with the gamification experience. Scores range from 0 (*complete dissatisfaction*) to 10 (*complete satisfaction*).

### 2.5. Data Analysis

Data were analyzed using SPSS Version 26, applying appropriate statistical tests based on variable type and distribution.

#### 2.5.1. Ethical Consideration

This study was approved by the ethical committee of the relevant university under code IR.VUMS.REC.1400.045. Students were informed about study objectives, and oral consent was obtained prior to data collection.

#### 2.5.2. Sample Participants

The study included 70 undergraduate nursing students, randomly assigned to two equal groups of 35. The mean age of participants was 19 years (SD = 0.9). Of the total, 51.4% (*n* = 36) were female and 48.6% (*n* = 34) were male. The average Grade Cumulative Score (GCS) was 15.71 (SD = 0.89) in the control group and 15.63 (SD = 0.83) in the intervention group, on a scale of 0–20. Results of an independent samples *t*-test showed no significant difference in GCS between the two groups (*t*(70) = 0.39, *p*=0.698).

## 3. Results

Table 1 presents the levels of knowledge in both groups before and after the intervention. An independent samples *t*-test revealed no significant difference in baseline knowledge between the control group (*M* = 6.83, SD = 2.02) and the intervention group (*M* = 6.3, SD = 1.94) (*t*(70) = 1.13, *p*=0.26). In contrast, after the 8-week internship, a significant difference was observed in knowledge scores between the control group (*M* = 9.58, SD = 2.93) and the intervention group (*M* = 13.39, SD = 1.64) (*t* (70) = 6.79, *p* ≤ 0.001).

In addition, results of another independent samples *t*-test indicated a significant difference in satisfaction levels at the end of the internship, with the control group scoring an average of 7.5 (SD = 1.34) and the intervention group scoring 8.7 (SD = 1.04, *t* (70) = 4.23, *p* ≤ 0.001).

Series of paired *t*-tests (Table 2) were conducted to compare the level of knowledge between the intervention group and control group before staring the internship (mean = 6.30  m, SD = 1.93) and at the end of 8 weeks (mean = 13.38, SD = 1.64), which showed a significant difference (*t*(35) = 35.29, *p* ≤ 0.001). We also compared the level of knowledge before and after the internship among controls. Similarly, this analysis also showed that there was a significant difference between levels of knowledge before and after the internship period (*t*(35) = 5.95, *p* ≤ 0.001).

In order to control the effect of basic knowledge among students who participated in gamification by Kahoot!, an ANCOVA (Table 3) was conducted. For this ANCOVA, the posttest score of knowledge was included as the dependent variable, two groups as the independent variable, and pretest score of knowledge as the covariate. Our results showed that the level of knowledge among students who played Kahoot! had significantly (*F* [1, 69] = 27.21, *p* ≤ 0.001) increased at the end of the 8-week internship, even after controlling for the effect of basic knowledge.

### 3.1. Item Analysis for Collaboration and Interest

In this questionnaire, “Interest” and “Flow” are treated as two distinct subcomponents of the broader construct of student engagement. “Interest” captures students' attention and curiosity toward the learning material, while “Flow” reflects deep immersion and enjoyment during the activity. These subscales are analyzed separately in accordance with “McCoy's [23] original validation.” Students' interest was measured using eight items on a five-point Likert scale, ranging from 1 (*strongly disagree*) to 5 (*strongly agree*). The questionnaire also includes two subscales: four items measuring interest in class and four items measuring flow. The final score is the sum of all eight items, ranging from 8 to 40, with higher scores indicating greater overall interest.  Table 4 presents item-level analysis for the intervention group (gamification).

Pertaining to the subfactor of flow, the highest answer was attributed to: 1: “I did not realize how time passed” (mean = 4.36) and 2: “I was completely absorbed in the activity” (mean = 4.36). Whereas, the lowest mean score was related to this item: “I enjoyed working on the tasks” (mean = 4.19). Overall, the mean score for the flow subscale was 17.19, with scores ranging from 14 to 20. For the interest subscale, the highest score was recorded for the item “They provided relevant feedback” (mean = 4.47). Whereas, the lowest mean score was associated with the item: “They provided exposure to new experiences” (mean = 4.03). Overall, the mean score for the “interest” subscale was 16.89 with a score range of 14–19.

A careful look at the item analysis (Table 4) for “collaboration” shows that the highest average is related to item number 5, “I communicated in a professional manner using respectful language” (mean = 4.42). The second highest average is attributed to item number 8, “I put in a lot of effort,” followed by item number 7, “Other group members communicated respectfully with me.” Nevertheless, the lowest average score was related to item number 1, “Brainstorming with fellow medical students was helpful” (mean = 3.69). Assessing the global score for the collaboration showed a mean of 34.10 (SD = 2.36) in a range of 26–37.

It should be noted that the variables of satisfaction, collaboration, and interest were measured only after the intervention among students who used Kahoot!. To explore these outcomes statistically, a one-sample *t*-test was conducted for each variable, comparing the group mean to the neutral midpoint value of 2.5. Accordingly, the average of all three variables of satisfaction (mean = 8.69, *t* = V, *p* ≤ 0.001), collaboration (mean = 33.47, *t* = 78.74, *p* ≤ 0.001), and interest (mean = 34.10, *t* = 93.18, *p* ≤ 0.001) was significantly higher than the average.

## 4. Discussion

Our findings indicated that gamification using the platform of Kahoot! effectively increased the level of nursing students' knowledge about vaccination after the end of the 8-week internship. This knowledge gain can be attributed to several internal factors specific to the intervention. One key mechanism was the real-time feedback embedded within the Kahoot! platform. Each session encouraged active learning through immediate correction of misconceptions, followed by instructor-led clarification. This form of feedback, combined with repeated exposure to vaccination content, supported deeper cognitive processing and long-term retention.

The use of gamification as a new teaching–learning strategy has attracted the attention of many researchers, and it should be noted that the use of this educational method is in accordance with the philosophy of active learning in nursing students. To the best of our knowledge, the effect of gamification specifically on vaccination in the community health internship program of nursing students has not been investigated. There is increasing evidence supporting the use of gamification in nursing education, including in community health nursing [15], cardiopulmonary resuscitation [21], and ECG interpretation [24], all of which have demonstrated an increase in students' knowledge. Consistently, Kahoot! was known to be a successful strategy to increase nursing students' knowledge regarding intramuscular injection [25]. Reviewing the literature revealed that nursing students considered gamification using Kahoot! very convenient, useful, and effective in formative evaluations and expressed that this educational strategy was helpful in preparation for initiating clinical courses [26].

Notwithstanding, there is evidence showing gamification using Kahoot! has no superiority to the Q&A method for improving the level of nursing students' knowledge regarding the topic of infection control [27]. These inconsistent findings can be explained by the wide usage and different implementation approaches that a serious game such as Kahoot! can be played. This is due to the fact that Kahoot! can be implemented individually, teamwork, before the beginning of the course for initial assessment, at the end of the course (summative assessment), and even as a continuous tool for formative assessment. Therefore, positive effects of using gamification to improve learning outcomes in nursing education imply the strong role of gamification as an additional teaching strategy [28].

The higher level of satisfaction reported by the intervention group also merits closer analysis. Unlike traditional internships, the gamified setting provided a low-stakes, enjoyable environment that promoted learner autonomy. Students could pace themselves, track their progress via the leaderboard, and experience friendly competition. Consistently, it has been shown that Kahoot! and Quizizz were more effective in satisfying nursing students when compared to Google Forms [29]. Higher levels of satisfaction among nursing students after gamification via Kahoot! are attributed to learners' engagement in the active learning process [30].

A deeper analysis of our findings reveals several key factors that may explain the positive impact of Kahoot! on students' satisfaction. First, the integration of mobile devices in the learning process appears to have significantly enhanced engagement. Many students demonstrate a strong preference for learning tools that incorporate smartphones and internet access, which align with their daily communication habits and digital fluency. Mobile-based platforms such as Kahoot! offer a familiar and accessible interface that supports active participation, particularly when students use their own devices to interact with the content and peers. Although this study did not assess students' digital literacy levels, the mobile-friendly design of Kahoot! likely facilitated ease of use, contributing to a more engaging and satisfying learning experience. Key gamification features, such as real-time interaction, audiovisual content, and instant scoring, helped to reinforce learning in a dynamic and user-centered environment [31].

Second, the platform's capacity to provide immediate and meaningful feedback played a critical role in enhancing learning satisfaction. As a web-based tool, Kahoot! promotes real-time formative assessment and encourages reflection on responses during gameplay. Prompt feedback is recognized by nursing students as a valuable component of learning, enabling them to monitor their progress and correct misunderstandings quickly [32, 33]. The opportunity to revisit key concepts through feedback reinforces learning outcomes and contributes to higher levels of satisfaction [34–36].

Third, the gamified structure actively fosters student interaction and collaboration. According to Walker et al. [37], satisfaction in nursing education is closely tied to interactive learning environments that strengthen both student–instructor and peer relationships. Kahoot!'s competitive and team-based dynamics stimulated classroom discussions and encouraged students to articulate their reasoning, brainstorm with peers, and reflect collectively. This informal, participatory atmosphere contrasts with traditional lecture-based learning, creating a more inclusive and engaging experience [38, 39].

Another key outcome of this study was the high level of student interest observed during the gamified sessions. It is important to distinguish between satisfaction and interest, while satisfaction reflects a general approval of the learning experience, interest is more closely linked to focused attention, curiosity, and emotional investment. In our study, indicators of student “flow,” such as losing track of time and full immersion in the activity, demonstrated that students were not merely enjoying the content but deeply engaged in it. Similarly, elements related to “interest,” such as the exposure to new learning experiences and the receipt of targeted feedback, were highly rated by participants. These findings align with previous literature indicating that the gamified learning experience using Kahoot! enhances students' intrinsic motivation and class participation [40]. This may be attributed to features such as competitive game mechanics, an enjoyable environment, and anonymous participation, which together reduce performance anxiety.

Our findings also indicated a strong sense of collaboration among students in the intervention group. Participants reported high levels of effort and mutual respect in classroom interactions. This aligns with the results of Dianati et al. [41], who found that students perceived Kahoot! as a user-friendly tool that supports collaborative learning through structured discussions, Q&A sessions, and brainstorming activities. Literature in the field suggests that teamwork skills are critical for students' future professional performance, and educational games serve as effective mediums for fostering communication, coordination, and collective problem-solving [42]. Overall, game-based strategies such as Kahoot! have been recognized for enhancing both student engagement and educator–student collaboration [30]. However, successful implementation depends largely on the instructor's willingness and comfort with using such tools, emphasizing the need for faculty development and institutional support [43].

### 4.1. Limitation

One limitation of this study is that, although applied knowledge related to practical skills (such as identifying injection sites and techniques) was assessed through the Kahoot! platform, direct observation of students' hands-on vaccination skills was not conducted. Future studies should incorporate objective structured clinical examinations (OSCEs) or direct practical assessments to comprehensively evaluate clinical skill performance.

### 4.2. Principal Results

The findings of this study indicate that gamification using Kahoot! can serve as a valuable supplementary educational tool for nursing students during internship programs. This approach supports the integration of clinical and theoretical content, which may lead to improved knowledge acquisition and overall performance. Future research is recommended to explore the effects of Kahoot!-based gamification in other nursing subjects focused on skill development, such as vital sign monitoring, injection techniques, and drug dosage calculation. In addition, evaluating the effectiveness of Kahoot! gamification among postgraduate nursing students would offer further insights into its broader applicability.

### 4.3. Comparison With Prior Work

To the best of our knowledge, this is the first study to examine the effect of Kahoot! on nursing students in the context of a community health course.

### 4.4. Conclusions

Overall, our findings demonstrate that nursing students' knowledge and satisfaction significantly increased following an 8-week gamification intervention using Kahoot!. Furthermore, levels of interest and collaboration among students were notably higher than the average benchmark score of 2.5, indicating a strong engagement with the gamified learning approach.

## Figures and Tables

**Table 1:** Comparing level of knowledge and satisfaction between the two groups, before and after intervention.

Variable	Intervention time	Group	Mean	SD	*t*	*p*
Knowledge	Before				1.13	0.26
		Control	6.83	2.02		
		Intervention	6.30	1.94		
	After				−6.79	≤ 0.001
		Control	9.58	2.93		
		Intervention	13.39	1.96		

Satisfaction	After				−4.23	≤ 0.001
		Control	8.7	1.34		
		Intervention	7.5	1.01		

**Table 2:** Comparing level of knowledge within the two groups before and after the internship.

Group	Intervention time	Mean	SD	*t*	*p*
Intervention	Before	30.60	193	−35.29	≤ 0.001
	After	38.13	1.64		

Control	Before	83.60	2.02	5.95	≤ 0.001
	After	58.90	2.93		

**Table 3:** ANCOVA test.

Predictors	Type III sum of squares	df	Mean square	*F*	Sig.
Intercept	311.13	1	311.13	75.72	< 0.001
Basic knowledge	111.79	1	111.79	27.21	≤ 0.001
Error	283.51	69	4.11		

**Table 4:** Item analysis of interest and collaboration.

Subfactor	Item	%	Mean	Max	Min
Interest			16.89		
1	They increased my interest in clinical practice	88.9	4.22	3	5
2	They added variety to the learning environment	86.2	4.17	3	5
3	They provided relevant feedback	94.5	4.47	3	5
4	They provided exposure to new experiences	77.8	4.03	3	5
Flow			17.19		
5	I did not realize how time passed	86.1	4.36	3	5
6	I enjoyed working on the tasks	83.3	4.19	3	5
7	I was completely absorbed in the activity	91.6	4.36	3	5
8	I found the tasks to be quite exciting	83.1	4.27	3	5
Collaboration			33.47		
1	Brainstorming with fellow medical students was helpful	61.1	3.69	2	5
2	Team discussion clarified concepts	83.4	4.22	2	5
3	Group decision making was useful	86.1	4.22	2	5
4	Working in a small team of 3 allowed better participation than working in a group of 10	72.2	3.97	3	5
5	I communicated in a professional manner using respectful language	94.4	4.44	3	5
6	I encouraged other members on the team to express their opinions	83.4	4.14	3	5
7	Other group members communicated respectfully with me	94.5	4.22	3	5
8	I put in a lot of effort	94.4	4.42	3	5

## Data Availability

The data supporting the findings of this study are available upon reasonable request from the corresponding author. Data are not publicly available due to privacy or ethical restrictions.
